# Supplementary material for: Topological protection by local support symmetry and destructive interference
Source: Nat Commun. 2026 Feb 13;17:2739. doi: 10.1038/s41467-026-69613-8 (PMC13013803; doi:10.1038/s41467-026-69613-8)
Supplement: Supplementary file 1 — Supplementary Information [file 41467_2026_69613_MOESM1_ESM.pdf]

Supplementary Information: Topological Protection by Local Support Symmetry and Destructive Interference

Jun-Won Rhim*

Department of Physics, Ajou University, Suwon 16499, Korea

Jaeuk Seo

Department of Physics, Korea Advanced Institute of Science and Technology, Daejeon 34141, Korea

Seongjun Mo

Department of Physics, Konkuk University, Seoul 05029, Korea

Hoonkyung Lee

Advanced Materials Program, Department of Physics, Konkuk University, Seoul, 05029, Korea

Sejoong Kim

Department of Electronic and Electrical Convergence Engineering, Hongik University, Sejong 30016, Republic of Korea

B. Andrei Bernevig†

Department of Physics, Princeton University, Princeton, New Jersey 08544, USA
Donostia International Physics Center, P. Manuel de Lardizabal 4, 20018 Donostia-San Sebastian, Spain and
IKERBASQUE, Basque Foundation for Science, Bilbao, Spain

I. MODEL-IV: DIRAC FERMIONS PROTECTED BY LOCAL SUPPORT C_{2y} SYMMETRY

We consider a three-site tight-binding model, as shown in Supplementary Fig. 1(a). The sublattices are denoted by A, B, and C. The Bloch Hamiltonian matrix is given by

$$\mathbf{H}(\mathbf{k}) = \begin{pmatrix} t_4 c_x + t_6 c_y & 1 + e_x + t_3(1 + e_x)\bar{e}_y & 0 \\ 1 + \bar{e}_x + t_3(1 + \bar{e}_x)e_y & t_5 c_y & t_2 + t_1 \bar{e}_x \\ 0 & t_2 + t_1 e_x & t_6 c_y \end{pmatrix}, \tag{1}$$

where $e_\alpha = \bar{e}_\alpha^* = e^{ik_\alpha}$ and $c_\alpha = \cos k_\alpha$ with $\alpha = x, y$. The hopping parameter between the neighboring A and B sites is set to be unity without loss of generality. Other hopping parameters are exhibited in Supplementary Fig. 1(a). One can note that the system respects C_{2x} if $t_3 = 1$. However, C_{2y} is always broken. As shown in the band structure plotted in Supplementary Fig. 1(b) and (c), the system exhibits band-crossings along XS although C_{2y} is absent. The dispersion relations along XS are evaluated as

$$E_1(\pi, k_y) = -t_4 + t_6 c_y, \tag{2}$$

$$E_2(\pi, k_y) = \frac{1}{2}\left((t_5 + t_6)c_y + \sqrt{4(t_1 - t_2)^2 + (t_5 - t_6)^2 c_y^2}\right), \tag{3}$$

$$E_3(\pi, k_y) = \frac{1}{2}\left((t_5 + t_6)c_y - \sqrt{4(t_1 - t_2)^2 + (t_5 - t_6)^2 c_y^2}\right), \tag{4}$$

where k_x is fixed to π. The corresponding eigenvectors are given by

$$\mathbf{v}_1(\pi, k_y) = (1, 0, 0)^{\mathrm{T}}, \tag{5}$$

$$\mathbf{v}_2(\pi, k_y) = c_2(k_y)(0, E_{sq,3}(\pi, k_y) - t_5 c_y, t_1 - t_2)^{\mathrm{T}}, \tag{6}$$

$$\mathbf{v}_3(\pi, k_y) = c_3(k_y)(0, E_{sq,2}(\pi, k_y) - t_5 c_y, t_1 - t_2)^{\mathrm{T}}, \tag{7}$$

where $c_2(k_y)$ and $c_3(k_y)$ are the normalization factors. The Hamiltonian matrix $\mathbf{H}$ is block-diagonalized at $k_x = \pi$ since $\mathbf{H}|_{12} = \mathbf{H}|_{21} = 0$. Namely, the model belongs to the trivial case (ii) in the main text, and A sites are completely isolated from B and C ones for $k_x = \pi$. This can be possible due to the destructive interference between amplitudes with staggered signs at A or B sites, as illustrated in Supplementary Fig. 1(d) to (f). Due to this property, the Bloch wave function corresponding to $\mathbf{v}_1$ spans only A sites as implied in the eigenvector formula in (5). On the other

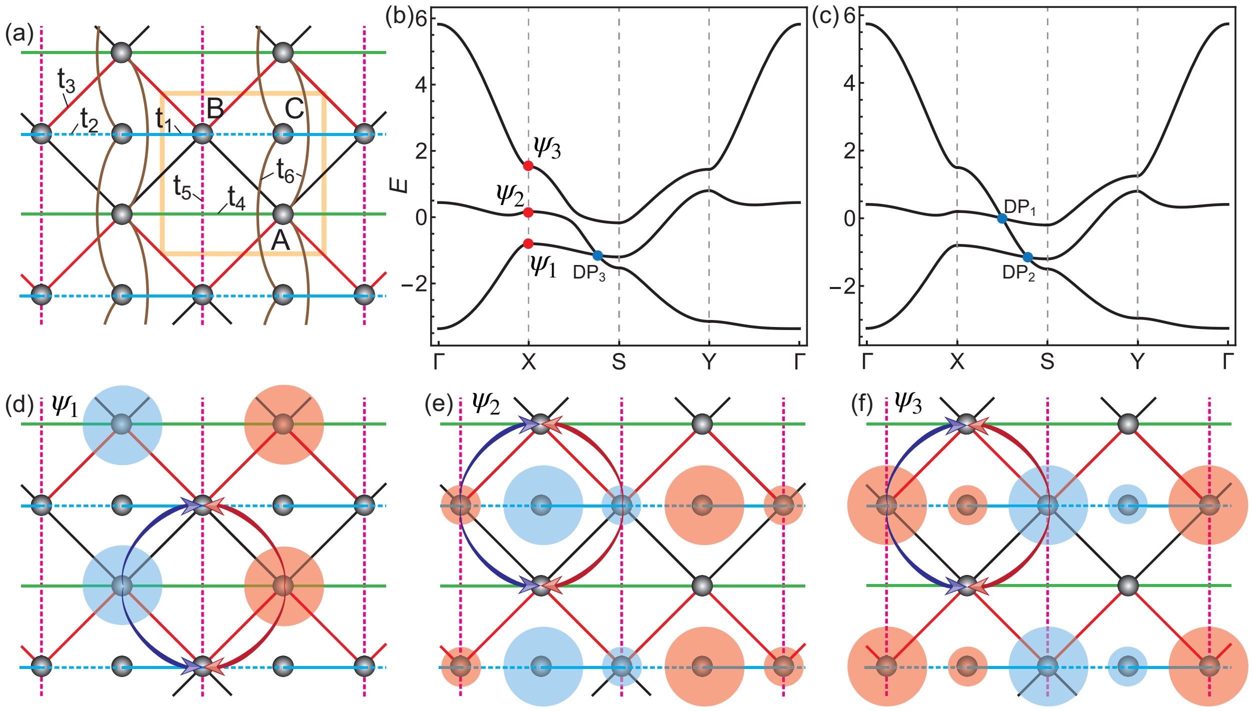

FIG. 1: (a) The hopping structure of the square lattice model-I. The unit cell is drawn by a yellow box, in which we denote three sublattices by A, B, and C. (b) and (c) plot band structures with tight-binding parameters $\{t_1, t_2, t_3, t_4, t_5, t_6\} = \{1, 1.2, 1, 1, 1.5, 0.2\}$ and $\{1, 1, 1, 1, 1.5, 0.2\}$, respectively. Dirac points are represented by DP_i. The high-symmetry points X, Y, and S stand for $\mathbf{k} = (0, \pi)$, $(\pi, 0)$, and (π, π), respectively. From (d) to (f), Bloch wave functions corresponding to Ψ_i's in (c) are illustrated. Their amplitudes are indicated by the colored circles, where the blue and red colors mean plus and minus signs, respectively. The size of the circles roughly implies the magnitude of the amplitude. The curved arrows explain how the hopping structure of the model hosts destructive interference to stabilize the compactly supported states.

hand, the Bloch wave functions corresponding to $\mathbf{v}_2$ and $\mathbf{v}_3$ span only B and C sites. As a result, the band-crossing point between $E_1(\pi, k_y)$ and other bands can be protected. Note that if $t_1 = t_2$, we have $E_1(\pi, k_y) = -t_4 + t_6 c_y$, $E_2(\pi, k_y) = t_5 c_y$, and $E_3(\pi, k_y) = t_6 c_y$ with corresponding eigenvectors $\mathbf{v}_1(\pi, k_y) = (1, 0, 0)^{\mathrm{T}}$, $\mathbf{v}_2(\pi, k_y) = (0, 1, 0)^{\mathrm{T}}$, and $\mathbf{v}_3(\pi, k_y) = (0, 0, 1)^{\mathrm{T}}$, respectively. In this case, three bands can have band-crossings with each other because they occupy different sites without any overlap. In the $t_1 = t_2$ case, the system respects mirror symmetry C_{2y}, and the Bloch wave functions corresponding to $\mathbf{v}_2$ is C_{2y} even(odd) while the other ones for $\mathbf{v}_1$ and $\mathbf{v}_3$ are C_{2y} odd(even) with respect to the symmetry axis $\hat{y}_1(\hat{y}_2)$. Therefore, the band-crossing points in the band structure, in this case, can also be understood from the mirror symmetry perspective.

II. FLUORINATED BIPHENYLENE NETWORK

We divide the system into $\mathcal{S}_1$ and $\mathcal{S}_2$ and denote sites belonging to them by A_i and U_i, respectively, as illustrated in Fig. 5(d) in the main text. Due to the fluorine atoms in the middle, C_{2y} symmetry is respected only in $\mathcal{S}_1$ while broken over the entire system. The Bloch Hamiltonian matrix of this system is given by

$$\mathbf{H}(\mathbf{k}) = \begin{pmatrix} \mathbf{h}_1(\mathbf{k}) & \mathbf{h}_{12}(\mathbf{k}) \\ \mathbf{h}_{12}^\dagger(\mathbf{k}) & \mathbf{h}_2(\mathbf{k}) \end{pmatrix}, \tag{8}$$

where

$$\mathbf{h}_1(\mathbf{k}) = \begin{pmatrix} 0 & t_4 & 0 & 0 & 0 & \bar{e}_x t_1 & 0 & 0 & e_y t_2 & 0 & 0 & \bar{e}_x e_y t_5 \\ t_4 & 0 & t_4 & 0 & 0 & 0 & 0 & 0 & 0 & 0 & 0 & 0 \\ 0 & t_4 & 0 & t_1 & 0 & 0 & 0 & 0 & 0 & e_y t_2 & e_y t_5 & 0 \\ 0 & 0 & t_1 & 0 & t_4 & 0 & 0 & 0 & 0 & e_y t_5 & e_y t_2 & 0 \\ 0 & 0 & 0 & t_4 & 0 & t_4 & 0 & 0 & 0 & 0 & 0 & 0 \\ e_x t_1 & 0 & 0 & 0 & t_4 & 0 & 0 & 0 & e_x e_y t_5 & 0 & 0 & e_y t_2 \\ 0 & 0 & 0 & 0 & 0 & 0 & 0 & 0 & t_4 & t_4 & 0 & 0 \\ 0 & 0 & 0 & 0 & 0 & 0 & 0 & 0 & 0 & 0 & t_4 & t_4 \\ \bar{e}_y t_2 & 0 & 0 & 0 & 0 & \bar{e}_x \bar{e}_y t_5 & t4 & 0 & 0 & 0 & 0 & \bar{e}_x t_1 \\ 0 & 0 & \bar{e}_y t_2 & \bar{e}_y t_5 & 0 & 0 & t_4 & 0 & 0 & 0 & t_1 & 0 \\ 0 & 0 & \bar{e}_y t_5 & \bar{e}_y t_2 & 0 & 0 & 0 & t_4 & 0 & t_1 & 0 & 0 \\ e_x \bar{e}_y t_5 & 0 & 0 & 0 & 0 & \bar{e}_y t_2 & 0 & t_4 & e_x t_1 & 0 & 0 & 0 \end{pmatrix}, \tag{9}$$

$$
\mathbf{h}_{12}(\mathbf{k}) = \begin{pmatrix}
t_6 & 0 & t_7 & 0 & t_8\bar{e}_x & 0 & 0 & 0 & 0 & 0 \\
t_3 & 0 & t_6 & t_6 & 0 & 0 & 0 & 0 & 0 & 0 \\
t_6 & 0 & 0 & t_7 & 0 & 0 & 0 & 0 & 0 & 0 \\
0 & t_6 & 0 & t_8 & 0 & 0 & 0 & 0 & 0 & 0 \\
0 & t_3 & 0 & 0 & t_6 & 0 & 0 & 0 & 0 & 0 \\
0 & t_6 & t_8 e_x & 0 & t_7 & 0 & 0 & 0 & 0 & 0 \\
0 & 0 & 0 & 0 & 0 & t_6 & t_6 & 0 & t_3 & 0 \\
0 & 0 & 0 & 0 & 0 & 0 & 0 & t_6 & 0 & t_3 \\
0 & 0 & 0 & 0 & 0 & t_7 & 0 & t_8\bar{e}_x & t_6 & 0 \\
0 & 0 & 0 & 0 & 0 & 0 & t_7 & 0 & t_6 & 0 \\
0 & 0 & 0 & 0 & 0 & 0 & t_8 & 0 & 0 & t_6 \\
0 & 0 & 0 & 0 & 0 & t_8 e_x & 0 & t_7 & 0 & t_6
\end{pmatrix}, \tag{10}
$$

and

$$
\mathbf{h}_{2}(\mathbf{k}) = \begin{pmatrix}
0 & 0 & t_4 & t_4 & 0 & 0 & 0 & 0 & 0 & 0 \\
0 & 0 & 0 & 0 & t_4 & 0 & 0 & 0 & 0 & 0 \\
t_4 & 0 & 0 & 0 & \bar{e}_x t_1 & t_2 & 0 & \bar{e}_x t_5 & 0 & 0 \\
t_4 & 0 & 0 & 0 & 0 & 0 & t_2 & 0 & 0 & 0 \\
0 & t_4 & e_x t_1 & 0 & 0 & e_x t_5 & 0 & t_2 & 0 & 0 \\
0 & 0 & t_2 & 0 & \bar{e}_x t_5 & 0 & 0 & \bar{e}_x t_1 & t_4 & 0 \\
0 & 0 & 0 & t_2 & 0 & 0 & 0 & 0 & t_4 & 0 \\
0 & 0 & e_x t_5 & 0 & t_2 & e_x t_1 & 0 & 0 & 0 & t_4 \\
0 & 0 & 0 & 0 & 0 & t_4 & t_4 & 0 & 0 & 0 \\
0 & 0 & 0 & 0 & 0 & 0 & 0 & t_4 & 0 & 0
\end{pmatrix}. \tag{11}
$$

Here, $\mathbf{H}(\mathbf{k})$ is written in the basis $\{|\mathrm{A}_1\rangle, \cdots, |\mathrm{A}_{12}\rangle, |\mathrm{U}_1\rangle, \cdots, |\mathrm{U}_{10}\rangle\}$ and $e_\alpha = \bar{e}^*_\alpha = e^{ik_\alpha}$. With the tight-binding parameters $\{t_1, t_2, t_3, t_4, t_5, t_6, t_7, t_8\} = \{-3.4, -3.1, -3.1, -3.2, -0.8, -0.06, 0.1725, 0.17\}$ we obtain the band spectrum in Fig. 5(b), which is similar to the DFT results in Fig 5(c).

The unitary matrix representing the local support C_{2y} operation for $\mathcal{S}_1$ given by

$$\mathbf{U}(\mathbf{k}) = \begin{pmatrix} \mathbf{u}_1(\mathbf{k}) & 0 \\ 0 & \mathbf{I}_{10\times 10} \end{pmatrix}, \tag{12}$$

where

$$\mathbf{u}_1(\mathbf{k}) = \begin{pmatrix}
0&0&1&0&0&0&0&0&0&0&0&0\\
0&1&0&0&0&0&0&0&0&0&0&0\\
1&0&0&0&0&0&0&0&0&0&0&0\\
0&0&0&0&0&\bar{e}_x&0&0&0&0&0&0\\
0&0&0&0&\bar{e}_x&0&0&0&0&0&0&0\\
0&0&0&\bar{e}_x&0&0&0&0&0&0&0&0\\
0&0&0&0&0&0&1&0&0&0&0&0\\
0&0&0&0&0&0&0&\bar{e}_x&0&0&0&0\\
0&0&0&0&0&0&0&0&0&1&0&0\\
0&0&0&0&0&0&0&0&1&0&0&0\\
0&0&0&0&0&0&0&0&0&0&0&\bar{e}_x\\
0&0&0&0&0&0&0&0&0&0&\bar{e}_x&0
\end{pmatrix}, \tag{13}$$

and $\mathbf{I}_{10\times 10}$ is a 10×10 identity matrix.

III. EDGE STATES OF THE MODIFIED LIEB LATTICE

In this section, we analyze the properties of the topological edge states of the modified Lieb lattice model introduced in the section "Model-I: topological insulator with local support time-reversal symmetry" of the main text. To this end, we consider a ribbon geometry of the model as illustrated in Fig. 2(a), which is translationally invariant along the y-axis while finite along the x-axis. The width of the ribbon is represented by the number of stripes (the yellow region), and the stripe indices are shown below the lattice structure in Fig. 2(a), from $n = 1$ to $n = N$. Therefore, the width of the ribbon in Fig. 2(a) is N. The unit cell is indicated by the gray box. Since we have four sites per stripe in the unit cell, there are $4N$ sites and $8N$ orbitals per unit cell, considering two spin species. As in the bulk system, A, B, and C sites belong to the $\mathcal{S}_1$ part while D sites

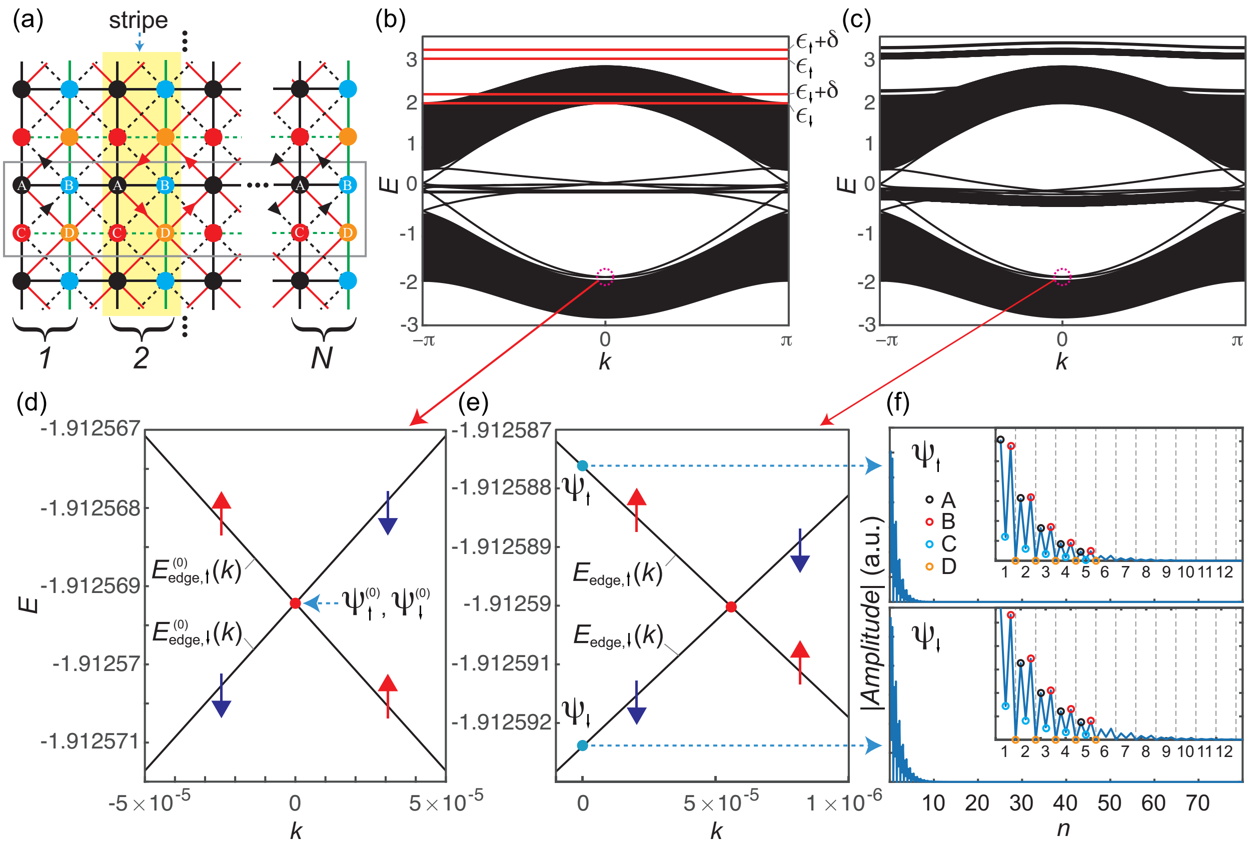

FIG. 2: (a) The ribbon geometry of the modified Lieb lattice model. The width N of this lattice is defined by the number of stripes along the x-axis. The yellow region indicates the $n = 2$ stripe. The gray box represents the unit cell of this ribbon geometry, in which we have $4N$ sites and $8N$ orbitals due to the spin degrees of freedom at all sites. (b) The band structure of the modified Lieb lattice with $N = 100$, where the inter-part coupling is turned off ($t = 0$). The onsite energies of spin-up and down at D-sites are $\epsilon_\uparrow$ and $\epsilon_\downarrow$, respectively. We apply additional onsite potential δ to the sites belonging to the edge dimer lines with $n = 1$ and $n = N$. The bands for states occupying the D sites are plotted by red lines. Here, we set $\alpha = 0.5$, $e_\uparrow = 3$, $e_\downarrow = 2$, and $t = 0$. (c) The band dispersion when the inter-part coupling t is turned on to 0.3. The band spectra in the dashed red circles in (b) and (c) are highlighted in (d) and (e). These bands host edge-localized states with different spin species, represented by red and blue arrows, and cross each other linearly, as indicated by red circles. The edge-localized band dispersions for $t = 0$ and $t = 0.3$ are expressed by $E^{(0)}_{\mathrm{edge},\uparrow\mathrm{or}\downarrow}(k)$ and $E_{\mathrm{edge},\uparrow\mathrm{or}\downarrow}(k)$, respectively. The Bloch states at $k = 0$ for the cases $t = 0$ and $t = 0.3$ are denoted by $\psi^{(0)}_{\uparrow\mathrm{or}\downarrow}$ and $\psi_{\uparrow\mathrm{or}\downarrow}$, respectively. (f) $|\psi_\uparrow|$ and $|\psi_\downarrow|$ in (e) are plotted as a function of the dimer line index n. These are highlighted in the insets. The empty circles with black, red, blue, and yellow colors represent A, B, C, and D sites, respectively.

consist of $\mathcal{S}_2$ part. The inter-part couplings are proportional to the model parameter t, and the time-reversal symmetry of the entire system is broken because the onsite energies $\epsilon_\uparrow$ and $\epsilon_\downarrow$ of the spin-up and down orbitals at D sites are unequal.

The band structure of a ribbon with width $N = 100$, where two parts $\mathcal{S}_1$ and $\mathcal{S}_2$ are uncoupled

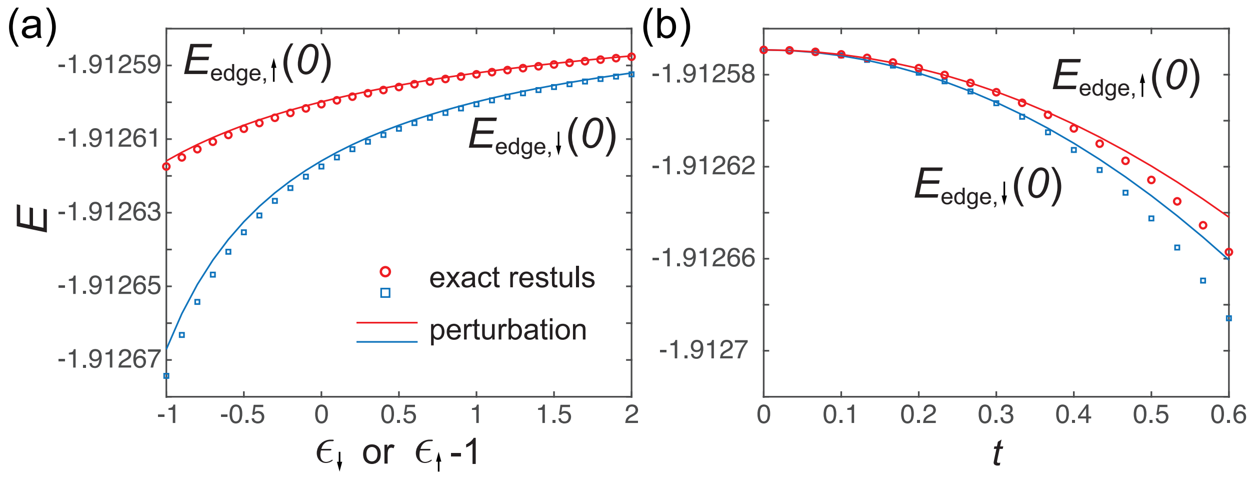

FIG. 3: In (a) and (b), we plot energies of the edge states at $k = 0$, $E^{(0)}_{\text{edge},\uparrow\text{or}\downarrow}(0)$ and $E_{\text{edge},\uparrow\text{or}\downarrow}(0)$, as a function of $\epsilon_\downarrow = \epsilon_\uparrow - 1$ and t, respectively. We compare exact calculations (empty circle and square symbols) and the results of the second-order perturbation method (solid lines).

($t = 0$), is drawn in Fig. 2(b). We use $\alpha = 0.5$, $e_\uparrow = 3$, and $e_\downarrow = 2$. We note that if the onsite energies are independent of the stripe index, Kramer's degeneracy of the edge bands in the red dashed circle is buried in the bulk continuum right below it. Therefore, we assign slightly different onsite potentials to the sites in $n = 1$ and $n = N$ stripes, which are larger than the onsite energies at the sites in the middle of the ribbon by an amount $\delta = 0.2$. Thus, we obtain the edge bands detached from the bulk continuum, as shown in Fig. 2(b). Spin-up and down electrons at D sites in $n = 1$ and $n = N$ stripes reside in the flat bands (red bands) at $\epsilon_\uparrow + \delta$ and $\epsilon_\downarrow + \delta$, respectively. On the other hand, the flat bands at $\epsilon_\uparrow$ and $\epsilon_\downarrow$ correspond to other spin-up and down electrons at D sites in the middle stripes ($2 \leq n \leq N - 1$). The band dispersion in Fig. 2(b) is deformed to Fig. 2(c) if the inter-part coupling t is turned on to 0.3. Since the electrons in the D sites can now hop to neighboring sites, the previously flat red bands acquire dispersion.

The crossings of two edge-localized bands in the red dashed circles in Fig. 2(b) and Fig. 2(c) are highlighted in Fig. 2(d) and Fig. 2(e), respectively. When $t = 0$, the edge states corresponding to the two bands in Fig. 2(d) are spanned solely by the orbitals in the $\mathcal{S}_1$ part. One can note that the spin-up band $E_{\text{edge},\uparrow}(k)$ and spin-down band $E_{\text{edge},\downarrow}(k)$ cross each other at the time-reversal invariant momentum $k = 0$. Even when we turn on the inter-part coupling, the crossing remains robust, as shown in Fig. 2(e). However, the band-crossing point shifts very slightly from the time-reversal invariant momentum. This is because edge modes are now coupled to the time-reversal broken part and obtain finite amplitudes for orbitals in D-sites. Nevertheless, the band-

crossing remains ungapped because there is no spin-mixing term in the Hamiltonian with inter-part coupling. It is noteworthy to remark that the size of the energy difference between two edge bands at $k = 0$ is the order of 10^{-5}, which is negligibly small compared with the magnitude of the inter-part coupling ($t = 0.3$). This is due to the extremely suppressed amplitude of orbitals at D-sites even after the coupling, as shown in the plot of the wave function in Fig. 2(f). Let us denote the unperturbed wave function of the edge bands at $k = 0$ as $|\psi_{\uparrow}^{(0)}\rangle$ and $|\psi_{\downarrow}^{(0)}\rangle$. Then, within the second-order perturbation method, the perturbed energy of the edge state is given by

$$
\begin{aligned}
E_{\text{edge},\sigma}(0) =& E^{(0)}_{\text{edge},\sigma}(0) + \langle\psi_{\sigma}^{(0)}|\mathbf{h}_{12}(0)|\psi_{\sigma}^{(0)}\rangle + \sum_{m}\frac{|\langle\psi_{\sigma}^{(0)}|\mathbf{h}_{12}(0)|\psi_{m,\sigma}^{(0)}\rangle|^2}{E^{(0)}_{\text{edge},\sigma}(0) - E^{(0)}_{m,\sigma}} \\
&+ \sum_{l}\frac{|\langle\psi_{\sigma}^{(0)}|\mathbf{h}_{12}(0)|\phi_{l,\sigma}^{(0)}\rangle|^2}{E^{(0)}_{\text{edge},\sigma}(0) - \epsilon^{(0)}_{l,\sigma}}, \qquad (14)\\
=& E^{(0)}_{\text{edge},\sigma}(0) + \sum_{l}\frac{|\langle\psi_{\sigma}^{(0)}|\mathbf{h}_{12}(0)|\phi_{l,\sigma}^{(0)}\rangle|^2}{E^{(0)}_{\text{edge},\sigma}(0) - \epsilon^{(0)}_{l,\sigma}}, \qquad (15)
\end{aligned}
$$

where $\sigma = \uparrow, \downarrow$, $\mathbf{h}_{12}(k)$ is the inter-part coupling term between $\mathcal{S}_1$ and $\mathcal{S}_2$, $\psi_{m,\sigma}^{(0)}$ is the Bloch states of the unperturbed model ($t = 0$) with momentum $k = 0$ and spin-σ belonging to $\mathcal{S}_1$ ($\psi_{m,\sigma}^{(0)} \neq \psi_{\sigma}^{(0)}$), and $\phi_{l,\sigma}^{(0)}$ is the Bloch states spanned by spin-σ orbitals in $\mathcal{S}_2$. We denote the energies corresponding to $\psi_{m,\sigma}^{(0)}$ and $\phi_{l,\sigma}^{(0)}$ as $E_{m,\sigma}^{(0)}$ and $\epsilon_{l,\sigma}^{(0)}$, respectively. From (14) to (15), we use the fact that the application of $\mathbf{h}_{12}$ to the states in $\mathcal{S}_1$ and $\mathcal{S}_2$ leads to ones belonging to $\mathcal{S}_2$ and $\mathcal{S}_1$, respectively. We note that the portion of orbitals at D-sites of the state given by $|\langle\psi_{\sigma}^{(0)}|\mathbf{h}_{12}(0)|\psi_{\sigma}^{(0)}\rangle|^2/t^2$ is extremely small, which leads to the hugely suppressed variation of the edge bands near the Kramer's point. In Fig. 3(a) and (b), we plot the energies of edge states at $k = 0$ as a function of $\epsilon_{\uparrow}$ and t, respectively, and note that the perturbation formula in (15) works well.

IV. HERRINGBONE LATTICE MODEL

The Herringbone lattice is illustrated in Fig. 4(a). There are four sites in the unit cell, indicated by a yellow box. These sites are denoted by A_1, B_1, C_1, and D_1. We assume that we have one orbital per site and consider only the nearest neighbor hopping processes with amplitude t_1. As shown in Fig. 4(a), the system possesses a nonsymmorphic rotation symmetry $g = \{C_{2y}|\frac{1}{2}\mathbf{a}_y\}$

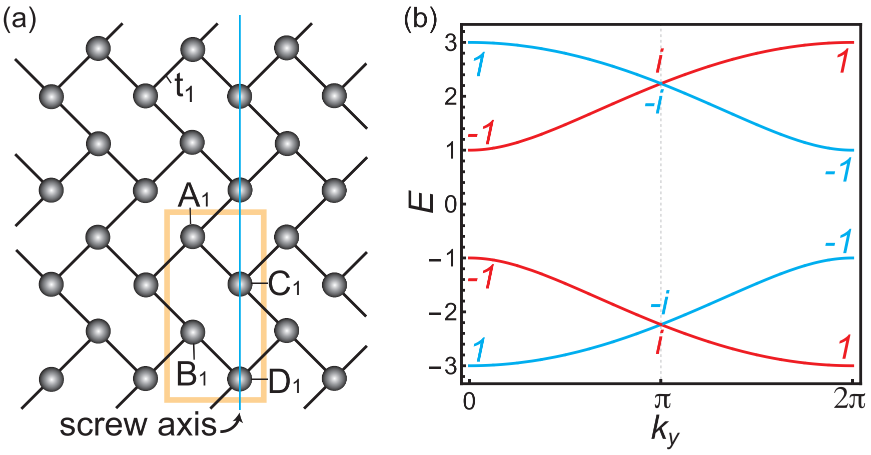

FIG. 4: (a) The herringbone lattice model with the nearest neighbor hooping processes with an amplitude t_1. The yellow box is the unit cell in which four sublattices, labeled by A_1, B_1, C_1, and D_1, exist. The screw axis is represented by a blue line. (b) The band dispersion for $t_1 = 1$ is plotted along k_y. The screw eigenvalues are displayed by colored numbers.

about the screw axis represented by a blue line. The Bloch Hamiltonian is given by

$$\mathbf{h}_1(\mathbf{k}) = t_1 \begin{pmatrix} 0 & 0 & 1+e^{-ik_x} & e^{ik_y} \\ 0 & 0 & e^{-ik_x} & 1+e^{-ik_x} \\ 1+e^{ik_x} & e^{ik_x} & 0 & 0 \\ e^{-ik_y} & 1+e^{ik_x} & 0 & 0 \end{pmatrix}. \tag{16}$$

The unitary matrix corresponding to the screw operation is given by

$$\mathbf{D}_{1,g}(\mathbf{k}) = \begin{pmatrix} 0 & e^{i(k_x+k_y)} & 0 & 0 \\ e^{ik_x} & 0 & 0 & 0 \\ 0 & 0 & 0 & e^{ik_y} \\ 0 & 0 & 1 & 9 \end{pmatrix}, \tag{17}$$

which satisfies

$$\mathbf{h}_1(g\mathbf{k}) = \mathbf{D}_{1,g}(\mathbf{k})\mathbf{h}_1(\mathbf{k})\mathbf{D}^{\dagger}_{1,g}(\mathbf{k}). \tag{18}$$

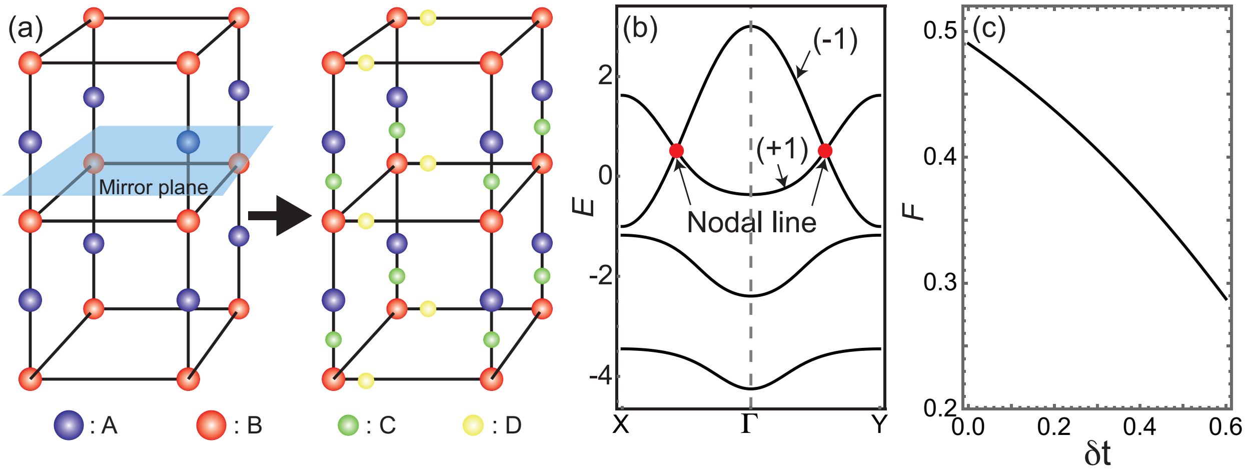

FIG. 5: (a) Lattice structure of the nodal-line semimetal model. The right panel shows the subsystem $\mathcal{S}_1$, with the mirror plane highlighted in blue. The left panel depicts the full lattice, including the $\mathcal{S}_2$ part composed of the C and D sites. (b) Band structure for $t_x = t_y = t_1 = u = \alpha = w = 1$, $t_z = -0.2$, and $\beta = 0.5$. The local-support mirror eigenvalues (± 1) of the two upper bands are indicated. (c) Robustness of the nodal line under perturbations δt that break the destructive-interference condition.

Along the symmetry-invariant momentum line, $\mathbf{k} = (0, k_y)$, the eigenenergies are evaluated as

$$E_{\alpha,\beta}(k_y) = \alpha\sqrt{5 + 4\beta \cos\frac{k_y}{2}}, \tag{19}$$

where $\alpha, \beta = \pm 1$. These bands are plotted in Fig. 4(b). The corresponding eigenvectors are obtained as

$$\mathbf{v}_{\alpha,\beta}(k_y) = c_{\alpha,\beta}(k_y)\begin{pmatrix} E_{\alpha,\beta}(k_y) \\ \beta E_{\alpha,\beta}(k_y)e^{-i\frac{k_y}{2}} \\ 2 + \beta e^{-i\frac{k_y}{2}} \\ 2\beta e^{-i\frac{k_y}{2}} + e^{-ik_y} \end{pmatrix}, \tag{20}$$

where $c_{\alpha,\beta}(k_y)$ is a normalization factor. One can show that the eigenvalue of $D_{1,g}(0, k_y)$ for $\mathbf{v}_{\alpha,\beta}(k_y)$ is evaluated as $\beta e^{-ik_y/2}$. Some values of $\beta e^{-ik_y/2}$ are displayed in Fig. 4(b).

V. 3D NODAL LINE SEMIMETAL MODEL

We propose a three-dimensional toy model that hosts a ring-shaped nodal line protected by a local-support mirror symmetry along the z-axis. We start from a subsystem, denoted by $\mathcal{S}_1$, which

possesses a global mirror symmetry with respect to the z-axis, as illustrated in Fig. 5(a). This subsystem is constructed on a cubic lattice containing two sites per unit cell. We assume that s-orbitals reside on these sites. The sub-Hamiltonian for $\mathcal{S}_1$, consisting of A and B sites, is given by

$$\mathbf{h}_1(\mathbf{k}) = \begin{pmatrix} 0 & f(\mathbf{k}) \\ f(\mathbf{k})^* & 0 \end{pmatrix}, \tag{21}$$

where

$$f(\mathbf{k}) = -t_x(1+e^{ik_z})\cos k_x - t_y(1+e^{ik_z})\cos k_y - t_z(1+e^{ik_z}) - it_1(1-e^{ik_z}). \tag{22}$$

We have a nodal line at $k_z = 0$ plane for $t_x = t_y = t_1 = 1$ and $t_z = -0.2$. This band-crossing is protected by the mirror symmetry with respect to the z-axis, whose mirror plane is indicated in Fig. 5(a). The mirror operation is represented by $M_z = \sigma_x$.

We now introduce two additional sites, labeled C and D, as shown on the right-hand side of Fig. 5(a). The subsystem composed of these two sites is denoted as $\mathcal{S}_2$. When $\mathcal{S}_1$ and $\mathcal{S}_2$ are coupled, the overall system no longer preserves the mirror symmetry. We assume that the C-site hosts an s-orbital, while the D-site is occupied by a p_z-orbital. Consequently, the hopping between the neighboring B and C sites vanishes, and the hopping amplitudes from the D-site to the adjacent upper and lower A-sites acquire opposite signs due to the odd parity of the p_z orbital. The sub-Hamiltonian for the $\mathcal{S}_2$ part is given by

$$\mathbf{h}_2(\mathbf{k}) = \begin{pmatrix} \epsilon_0 & \alpha + \beta e^{-ik_z} \\ \alpha + \beta e^{ik_z} & \epsilon_0 \end{pmatrix}, \tag{23}$$

where we set $\epsilon_0 = -2$, $\alpha = 1$, and $\beta = 0.5$. On the other hand, the inter-part Hamiltonian is given by

$$\mathbf{h}_{12}(\mathbf{k}) = \begin{pmatrix} u & 0 \\ u & w(1-e^{ik_z}) \end{pmatrix}, \tag{24}$$

where the second column reflects the properties of hopping processes between the p_z orbital at the D site and s orbitals at A and B sites, which are mentioned above. According to the gen-

eral theory for semimetal cases in the maintext, the columns of the inter-part Hamiltonian should be proportional to the eigenstate of the symmetry operation with an eigenvalue 1 or vanish at symmetry-invariant momenta for the nodal line to be protected although mirror symmetry is broken. In this nodal line semimetal model, the inter-part coupling indeed fulfills these conditions. The first column of $\mathbf{h}_{12}(\mathbf{k})$ is proportional to $(1,1)^{\mathrm{T}}$, which is the eigenvector of M_z with an unity eigenvalue, and the second column vanishes at $k_z = 0$. Consequently, the nodal line between the two bands of $\mathbf{h}_1(\mathbf{k})$ at $k_z = 0$ remains intact even when the parameters u and w are switched on, as shown in Fig. 5(b). The two crossing bands carry opposite local-support mirror eigenvalues, and the eigenstates with mirror eigenvalue -1 exhibit compact localization, having vanishing amplitudes on the C and D sites. Such eigenstates are stabilized through destructive interference: the opposite amplitudes on the A and B sites cancel at the C site, ensuring that the wave function remains confined within $\mathcal{S}_1$. To quantify the robustness of the nodal line, we compute its fragility, defined as $\Delta/\delta t$, against an additional hopping term that breaks the local-support condition by modifying the $(2,2)$ element of $\mathbf{h}_{12}(\mathbf{k})$ to $w(1-e^{ik_z})+\delta t$. Here, Δ denotes the maximum gap opened along the nodal line. As shown in Fig. 5(c), the fragility decreases as δt increases, since the induced gap grows sublinearly as a function of δt.

---

* Electronic address: jwrhim@ajou.ac.kr

† Electronic address: bernevig@princeton.edu
